# Factors Influencing Willingness to Share Health Misinformation Videos on the Internet: Web-Based Survey

**DOI:** 10.2196/30323

**Published:** 2021-12-09

**Authors:** Alla Keselman, Catherine Arnott Smith, Gondy Leroy, David R Kaufman

**Affiliations:** 1 Office of Engagement and Training National Library of Medicine Bethesda, MD United States; 2 Information School University of Wisconsin Madison Madison, WI United States; 3 Management Information Systems University of Arizona Tucson, AZ United States; 4 Medical Informatics Program SUNY Downstate Health Sciences University Brooklyn, NY United States

**Keywords:** misinformation, information literacy, science literacy, webcasts as topic, YouTube

## Abstract

**Background:**

The rapidly evolving digital environment of the social media era has increased the reach of both quality health information and misinformation. Platforms such as YouTube enable easy sharing of attractive, if not always evidence-based, videos with large personal networks and the public. Although much research has focused on characterizing health misinformation on the internet, it has not sufficiently focused on describing and measuring individuals’ information competencies that build resilience.

**Objective:**

This study aims to assess individuals’ willingness to share a non–evidence-based YouTube video about strengthening the immune system; to describe types of evidence that individuals view as supportive of the claim by the video; and to relate information-sharing behavior to several information competencies, namely, information literacy, science literacy, knowledge of the immune system, interpersonal trust, and trust in health authority.

**Methods:**

A web-based survey methodology with 150 individuals across the United States was used. Participants were asked to watch a YouTube excerpt from a morning TV show featuring a wellness pharmacy representative promoting an immunity-boosting dietary supplement produced by his company; answer questions about the video and report whether they would share it with a cousin who was frequently sick; and complete instruments pertaining to the information competencies outlined in the objectives.

**Results:**

Most participants (105/150, 70%) said that they would share the video with their cousins. Their confidence in the supplement would be further boosted by a friend’s recommendations, positive reviews on a crowdsourcing website, and statements of uncited effectiveness studies on the producer’s website. Although all information literacy competencies analyzed in this study had a statistically significant relationship with the outcome, each competency was also highly correlated with the others. Information literacy and interpersonal trust independently predicted the largest amount of variance in the intention to share the video (17% and 16%, respectively). Interpersonal trust was negatively related to the willingness to share the video. Science literacy explained 7% of the variance.

**Conclusions:**

People are vulnerable to web-based misinformation and are likely to propagate it on the internet. Information literacy and science literacy are associated with less vulnerability to misinformation and a lower propensity to spread it. Of the two, information literacy holds a greater promise as an intervention target. Understanding the role of different kinds of trust in information sharing merits further research.

## Introduction

### Introducing the Concern

When it comes to digital consumer health information, web-based resources can provide both valuable information and misinformation [1]. Concerns regarding the quality of health information and individuals’ susceptibility to misinformation are as old as health information websites that target consumers. The changing digital ecosystem of the social media era has increased the potential for and the speed of spread of misinformation, making these concerns more urgent. At present, web-based health information exists in many different formats, often with videos and interactive features. Entire networks, such as YouTube, enable the sharing of videos in an easy manner accessible to a broad public. Social networks allow consumers to spread links to sources that promise quick and easy remedies and promote wellness approaches that are not supported by evidence.

A common theme in non–evidence-based consumer health information is strengthening the immune system and developing natural immunity against infectious diseases. This topic relates to many important public health concerns, from vaccination compliance to individual behavior during epidemics. The COVID-19 pandemic and the need for pandemic-curbing measures underscore the importance of understanding how the public evaluates and shares information about non–evidence-based remedies. We are writing this paper approximately a year after the pandemic reached the United States, and the word *infodemic* is often used to characterize the accompanying epidemic of unreliable information about the virus and the disease [2].

An extensive body of research suggests that individuals often lack the information skills required for navigating effectively through a multitude of health information sources of varying formats and quality [3]. To the best of our knowledge, no studies to date have attempted to characterize the relationship between an individual’s tendency to share or recommend a video with non–evidence-based health advice and multiple cognitive and social factors. Understanding how such factors may affect information sharing is essential to curtail the spread of misinformation. This study is concerned with the types of statements that individuals view as convincing support of health claims, as well as the relationship between several factors—information literacy, science literacy, knowledge of the immune system, interpersonal trust, and trust in health authority—and inclination to share a non–evidence-based claim.

### A Word on Misinformation

This study focuses on a YouTube video that promotes a dietary supplement, making strong claims about this supplement’s ability to *boost* the immune system. Research into how vitamins, minerals, enzymes, and other substances may modulate the immune system is highly complex. However, the claims made by the video are simple and confident. While discussing these claims, we use the term *misinformation*, hence not making any assumptions about the intent of the supplement’s producers and promoters.

Misinformation has been defined as *objectively incorrect information that is not supported by scientific evidence and expert opinion* [4]. Although the 2016 US presidential election brought intense attention to the phenomenon, Molina et al [5] analogized it to a much older problem—*yellow journalism*—dating from the Spanish-American War of 1896. It differs from disinformation in that disinformation connotes “an intentional, deliberate, or purposeful effort to mislead, deceive, or confuse.”

Wardle and Derakhshan [6] considered information problems for journalists and outlined 7 types of misinformation and disinformation. In this taxonomy, problematic content is contrasted with genuine content:

1. Misleading content (misleading use of information)
2. Satire or parody (without intention to harm)
3. Fabricated content (false content intended to deceive)
4. Imposter content (impersonation of genuine sources)
5. Manipulated content (manipulation of genuine sources to deceive)
6. False context (attached to genuine content)
7. False connection (mismatch of headline to content with intent to deceive).

Clearly, some of these problematic content types are more malicious than others. However, whether intentional or not, misinformation can cause harm. This paper focuses on health information that makes unsubstantiated and exaggerated claims, purposefully or not, with the ultimate objective being to promote and sell a product.

### Health Misinformation on the Internet

The prevalence of health misinformation is an ongoing concern. Keselman et al [7] analyzed 24 type 2 diabetes top result pages, obtained from a Google search on *diabetes*, *reversal*, and *natural*. Most of the sites either promised or implied full recovery, most commonly achieved by taking dietary supplements, making claims that opposed the evidence-based perspective of the American Diabetes Association.

Research into behavior on the internet shows that people are susceptible to misinformation and are willing to share it. Web-based health misinformation has been investigated in various places, including web-based health communities [6], social media [8,9], Google Trends [10,11], Amazon’s web-based bookstore [12], and smart assistants such as Apple Siri and Amazon Echo [13].

Most research into how people spread misinformation in web-based social networks has been conducted in the domains of vaccines and infectious diseases [14]. For example, Basch et al [15] analyzed the most viewed vaccine-related videos on YouTube. The most popular videos were antivaccine (65.5%). They were commonly posted by laypeople and most often focused on the causal links between vaccines and autism. This and other such studies [16,17] not only illustrate the prevalence of misinformation but also underscore its potential impact. Krishna found that “knowledge-deficient, vaccine-negative individuals” were more active in spreading messages on the internet about vaccines [18]. The messages they spread were aligned with their attitudes.

In a study that focused on social media spread, Chen et al [19] analyzed 2691 tweets on Weibo (a Chinese equivalent of Twitter) related to breast and cervical cancers. They found that about 30% of the tweets contained misinformation, and that treatment-related tweets were more likely to contain misinformation than prevention-related tweets.

Sommariva et al [8] investigated stories circulating in social networking services about the Zika virus and found that half of the *top 10* were either misinformation or rumors. Studies are now beginning to emerge, focusing on the exchange of COVID-19 misinformation. Two-thirds of Korean adults surveyed by Lee et al [20] reported encountering myths and misinformation about COVID-19. Scientific discoveries, especially when they are of focal public interest and rapidly disseminated, beget misinformation; COVID-19 vaccines are being developed so fast that acquiring enough information about them to make decisions is a difficult task. In the United States, in May 2020, 73% of Republicans and 82% of Democrats surveyed called COVID-19 misinformation “a major problem,” and almost 70% of those surveyed identified social media as the principal source of such misinformation [9].

### Information Competencies

#### Overview

Researchers have been looking for effective approaches to promote accurate information in impactful ways and for factors that may reduce individual vulnerability. One of the most researched factors is health literacy, sometimes defined as “the degree to which individuals have the capacity to obtain, process, and understand basic health information and services needed to make appropriate health decisions.” [21]. Other cognitive factors, while emerging as important theoretical constructs, have been studied less often in an empirical context. Sometimes these constructs are merged with the concept of health literacy (for a review of the conceptual underpinnings and research gaps in health literacy [22,23]). Three of these constructs—information literacy, science literacy, and trust—were considered in this study.

#### Information Literacy

Pinto et al reviewed the evolution of information literacy as a term and concept [24]. One useful definition is that information literacy is “a set of aptitudes to locate, handle, evaluate and use information efficiently for a wide variety of purposes, generating competent citizens who are better able to perform their activities in the new society” [24].

Information literacy instruction originated in libraries, in the practice known as *bibliographic instruction*. For this reason, both instruction and evaluation techniques were developed for *static, primarily print, sources* [24] and not the information content encountered by today’s social media users. For this reason, the assessment of information literacy is typically conducted by academic librarians engaged in the instruction of a specific community of learners—from kindergarten through college—to determine that those learners are meeting the goals of a specific library instruction program. Instruments are developed by librarians and for libraries to fit their institutional missions. Information literacy is an *essential learning outcome* according to the Association of American Colleges and Universities [25].

Information literacy skills apply to any domain of information, including but not limited to health. An information-literate person is one who has “learned how to learn” [26]. That person can find appropriate information resources to meet their specific needs, can search those information resources effectively to find information, and distinguish between different types of resources based on characteristics that include quality markers. Health literacy, in contrast, relates specifically to outcomes affecting the health of the individual, for example, the ability to function in the health care environment (Berkman et al [27] presents a review of health literacy definitions).

Research assessing the effectiveness of information literacy is difficult to find, in part, because there are *very few* measures of the application of real-world learning [28]. However, the importance of information literacy for effectively navigating information and avoiding misinformation is in the very definition of the concept of information literacy.

#### Science Literacy

Researchers in the field of science education describe scientific literacy as comprising 3 components: (1) knowledge of science, (2) knowledge about science, and (3) attitudes toward science [29].

Knowledge of science refers to understanding specific scientific concepts, for example, being able to explain how antibodies bind to antigens. Research into the impact of knowledge of science on daily life has been very limited, and the few existing studies have produced mixed results. Layton et al [30] studied how people make decisions in situations where science has a potential bearing, for example, the older adults planning their heating budgets. They found that people rarely framed their problems as scientific ones, attending more to social and emotional aspects (eg, the pleasure of a glowing heater). In contrast, Keselman et al [31] found that the depth of biological knowledge contributed to adolescents’ ability to reject myths about HIV. Overall, there is no convincing evidence that the knowledge of science is a ubiquitous and powerful aid in real-life situations. In part, the uncertain role of knowledge may be attributed to the depth of knowledge required. More recently, Keselman et al [7] evaluated websites promoting remedies for type 2 diabetes and found that the treatment mechanisms described on these sites referred to concepts well beyond high school biology.

Knowledge about science refers to understanding what constitutes a scientific question, how science may go about deriving the answer, and the checks and balances that exist to minimize bias and reduce errors [29]. Knowledge about science is a potentially promising competency that may be related to evaluating health information on the internet. Individuals with this competency may be skeptical about conspiracy claims and promises of simple solutions for complex problems and attentive to egregious research design flaws. Although science education recognizes the importance of knowledge about science [32,33], empirical investigations are complicated by the absence of operational definitions and assessment tools. Few instruments isolate this component from general science literacy; the one instrument we found (and used as a basis for this study) targets students in college-level science classes [34].

The third component, attitude toward science, incorporates the view that science is valuable as an advantageous way to derive knowledge about the natural world: understanding the importance of the scientific process and trusting science as an enterprise. Trust in science can undeniably influence individuals’ beliefs, consequential to matters of health. Agley and Xiao conducted a cross-sectional survey on the believability of 5 COVID-19 narratives, ranging from *scientific consensus* to *conspiratorial* [35]. Trust in science was the only significant predictor of the acceptance of more scientifically grounded narratives. Jennings et al [36] also found that trust played a role in the willingness to get vaccinated and the rejection of conspiracies.

While discussing science literacy throughout the rest of this paper, we use the term to refer to the *knowledge about science* component, as a generalizable aspect of science literacy that can be addressed through science education. When we write about knowledge of science, we refer to knowledge of the immune system, as the area of science knowledge that is directly applicable to the subject of our study.

In everyday situations that require weighing evidence, science literacy may be overridden by other factors. One factor is sociocultural. The cultural cognition thesis [37] proposes that individual perceptions of societal risks cohere with values characteristic of groups with which they identify. People process information in a manner adjusted to preserve identity-consistent rather than factually correct beliefs [36]. In a large-scale study evaluating views on climate change, Kahan et al [38] found that participants with the most sharply polarized cultural views, not those with high scientific literacy, were most concerned about climate change.

Pennycook et al [39,40] put forth a different kind of argument. They draw on dual-process theory [41] to distinguish System 1 thinking—fast, intuitive, and effortless—from System 2 thinking—analytic and effortful. Pennycook et al [42] argue that lazy System 1 thinking, more than strong partisan belief, predisposes individuals to believe in fake news. People tend to share false health claims from social media partly because they fail to sufficiently consider whether the content is accurate [42]. Moreover, different information presentation formats tend to trigger different systems, with film, compared with graphs and formulas, being more likely to activate analytical reasoning [43].

#### Trust

In social science literature, the concept of interpersonal trust is a measure of social capital that reflects the goodwill and sense of community that members of a society feel toward one another [44]. Higher general trust is a sign of well-functioning democratic societies and cohesive communities [45]. Multiple studies have found that trust is positively correlated with self-reported health and happiness [45]; trust is viewed as a positive, desirable characteristic of individuals in societies. Generalized trust also correlates with institutional trust in the government and its foundational institutions. Rotter distinguished between *trust* and *gullibility* by defining trust as “believing others in the absence of a clear-cut reasons to disbelieve” [46]. However, to the best of our knowledge, no study has investigated the relationship between interpersonal trust and web-based information behavior. In developing this study, we were interested in understanding the relationship among interpersonal trust, trust in health authorities (eg, leading biomedical research organizations, public health authorities, health care providers) and susceptibility to web-based health misinformation.

### Objectives

The study described in this paper has 3 specific objectives:

1. Assess individuals’ willingness to share a non–evidence-based YouTube video about strengthening the immune system and characterize types of evidence that positively or negatively affect the opinion of a non–evidence-based remedy.
2. Describe levels of information literacy, science literacy, knowledge of the immune system, and trust among the participants.
3. Characterize the relationship between information literacy, science literacy, knowledge of the immune system, trust, and the likelihood of recommending a YouTube video about a supplement with an immunity-boosting claim.

## Methods

### Participants

Participants (N=150) were recruited via Regional Medical Libraries (RMLs) of the National Network of the Libraries of Medicine (NNLM). NNLM is a diverse network of 7186 academic health sciences libraries, other special biomedical libraries, public libraries, information centers, and community-based organizations. RMLs coordinate the Network’s operations in their regions, supporting and coordinating regional and national programs focused on health information. One coauthor emailed the 8 RML Associate and Executive Directors in late November of 2020 and requested permission to send out the study recruitment announcement to public libraries and community organizations using the listservs in their networks. Listserv members—employees of NNLM member organizations—were asked to promote the survey link among their patrons or clients via means appropriate to their environment. We hoped that this recruitment method would increase the likelihood of the geographic and cultural diversity of the participants. The announcement specified that participants needed to be proficient in English and comfortable using the internet. Professional librarians were excluded from the study.

The initial objective was to recruit 100 participants. Although we did not have previous data that could serve as the basis for power analysis to estimate the needed sample size, past experience suggested that this number would provide a sufficiently large sample size to answer our inferential questions; however, it would also be small enough for a thorough review of open-ended questions that would enrich the study. The first response was received on December 2. On December 3, the survey was closed because the target number had been reached.

Participants came from 34 US states, including Alaska and Hawaii, and from the District of Columbia. Demographic characteristics of the participants are presented in Table 1.

Upon completing the survey, participants received a US $50 Amazon e-gift card. The study protocol was exempted from Institutional Review Board’s review by the National Institutes of Health Office of Human Subjects Research.

**Table 1:** Demographic characteristics of the participants (N=149)^a^.

Demographic characteristics			Participants, n (%)
**Age (years)**			
	18-29	35 (23.5)	
	30-49	87 (58.4)	
	50-64	24 (16.1)	
	65+	3 (2)	
**Education**			
	High school or less	12 (8.1)	
	Some college	28 (18.8)	
	College graduate	83 (55.7)	
	Postgraduate degree	26 (17.4)	
**Gender**			
	Female	70 (47)	
	Male	79 (53)	
**Race and ethnicity**			
	White	89 (59.7)	
	Black or African American	21 (14.1)	
	Hispanic or Latino	17 (11.4)	
	Asian	5 (3.4)	
	American Indian or Alaska Native	10 (6.7)	
	Native Hawaiian or Other Pacific Islander	2 (1.3)	
	Declined to answer	7 (4.7)	
**Residential setting**			
	Urban	102 (68.5)	
	Rural	11 (7.4)	
	Suburban	36 (24.2)	

^a^One participant did not complete the demographic section.

### Instruments

Participants completed a web-based survey comprising the following components (Multimedia Appendix 1).

#### Web-Based Health Information Evaluation Task

For this task, participants viewed a 5-minute YouTube video, promoting a dietary supplement to strengthen the immune system [47].

Participants received the following prompt:

Your cousin had a bad case of the flu last year and did not fully recover for several weeks. In recent years, your cousin has frequently suffered from cold and occasionally flu. A friend told you about how certain supplements can help rev up your immune system. That friend recommends the following video: (link to the video).

The video is an excerpt from the 2019 TMJ4-TV News episode of *The Morning Blend* TV show, in which 2 anchors talk to a Welltopia Pharmacy pharmacist about a cold and flu-fighting dietary supplement Viracid, which is claimed to “boost your immune system like no other.” The episode is sponsored by Welltopia Pharmacy, and the sponsorship is clearly indicated in the video caption. The studio table contains multiple bottles of Viracid. On the close-up, viewers can see the price tag of US $37.50 on a bottle with 60 capsules. The pharmacist recommends taking one capsule of the supplement every hour at the onset of cold and flu symptoms. The pharmacist claims that the ingredients are “very pure,” “in concentrated form,” and “the best immune booster” on the market. The video offers a 10% discount when buying the supplement.

After viewing the video, the participants answered several questions. The first asked them how likely they were to recommend the video to their cousin. The 4-point Likert score answers ranged from *Very unlikely* to *Very likely*. Participants were also asked to explain their decision and comment in free text on the convincing and concern-raising aspects of the video. Next, they were presented with several hypothetical pieces of evidence and asked how likely that evidence was to affect their opinion about the supplement. The answer options were *Very likely, negatively*; *Somewhat likely, negatively*; *No effect*; *Somewhat likely, positively*; and *Very likely, positively*. The hypothetical pieces of evidence are as follows:

- A friend taking this supplement for the past 2 years had not had a cold.
- The supplement’s rating on a crowdsourced review site is 4.5/5.
- The supplement’s rating on a crowdsourced review site is 2.3/5.
- A survey of consumers of the product reported that 85% did not have the flu within a year.
- Positive reviews on the supplement producer’s website.
- The explanation of the supplement’s biochemical mechanism of action on the company’s website.
- Knowledge that the supplement has not been evaluated in a clinical trial.
- The National Institute of Allergy and Infectious Diseases (NIAID) review finding inconclusive evidence about the effectiveness of the supplement.
- NIAID review finding evidence of the effectiveness of the supplement in reducing the frequency and severity of respiratory infections.

Participants had the opportunity to explain their ratings.

#### Demographic Survey

The demographic survey included questions about participants’ gender, age, race and ethnicity, highest level of education achieved, and place of residence.

#### Information Literacy Survey

The information literacy survey, developed by the authors, comprised 6 multiple-choice questions. The questions were modeled on skill domains and questions presented in 2 existing instruments: *Test of Scientific Literacy Skills* (TOSLS) [34] (refer to the *Science Literacy Survey* section) and *Parenting Plus Skills Index* [48]. The *Parenting Plus Skills Index* was validated in 3 samples of 500 Australian adults, ages 20-44 years. In addition, McKenzie’s checklist for evaluating health information [49], which is not an instrument but a decision aid intended for health educators to share with consumers, was used to further support the choice of particular domains of skills to test. The internal consistency of the survey, measured by Cronbach α, was 0.62 considered acceptable [50].

#### Science Literacy (Knowledge About Science) Survey

This survey, which comprised 12 multiple-choice questions, was developed based on TOSLS, with a number of modifications. TOSLS, which measures the scientific literacy skills of students in undergraduate biology classes, includes questions that assess competency in 9 scientific literacy skills. Of these 9, we adopted 2, *identifying a valid scientific argument* and *understand elements of research design and how they impact scientific findings or conclusions*, as highly relevant to evaluating the content of web-based health claims. We excluded skills that pertained to conducting first-order research inquiry and analyzing and presenting quantitative data (*solve problems using quantitative skills, including probability and statistics*), as we saw these as more relevant to performance in undergraduate biology classes than to consumer health tasks. We also excluded *evaluate the validity of sources*, viewing this as a key information literacy competency, measured by our information literacy test. Finally, we excluded questions that involved judging the appropriateness of the *use of science by government, industry, and media*.

To develop our measure, we adopted the structure and content of some questions and answer options by Gormally et al [34], while simplifying the language (as our primary audience was not a college class) and editing the content to make it applicable to the health domain. We also wrote several additional health-specific questions and answer options that pertained to the 2 scientific literacy skills relevant to our task. The resulting measure comprised 12 questions, 5 assessing the ability to identify a valid scientific argument, and 7 assessing understanding of research design and how it pertains to scientific findings.

TOSLS was developed through an iterative process using built-in validation procedures. According to Gormally et al [34]:

...measures of validity included correspondence between items and scientific literacy goals of the National Research Council and Project 2061, findings from a survey of biology faculty, expert biology educator reviews, student interviews, and statistical analyses.

Although TOSLS validity data do not extend to our measure, TOSLS provides a theoretical foundation for our conceptualization of scientific literacy as it pertains to evaluating web-based health information. The items were developed through several iterative review and discussion rounds by the 4 authors. The internal consistency of our science literacy survey, measured by Cronbach α, was 0.6, which is considered acceptable [50].

#### Immune System Knowledge Survey

We devised a five-question immunology knowledge assessment test to measure the basic knowledge of the immune system. The questions were developed with the expectation that anyone who had taken a middle or high school biology course could answer them correctly. The issue is whether someone with basic knowledge of the immune system would assess the claims expressed in the video from a more critical vantage point. Immunology is an immensely complex topic that addresses issues well beyond the scope of our study.

#### Interpersonal Trust Survey

This survey assessed interpersonal trust and included one question commonly used in interpersonal trust surveys [51]. The question asked, “Generally speaking, would you say that most people can be trusted or that you cannot be too careful in dealing with people?” The answers required choosing a number on the scale from 1 to 5, where 1 meant *you can’t be too careful* and 5 *most people can be trusted*.

#### Trust in Health Authority Survey

This survey, developed by the researchers, assessed participants’ trust in 5 established authoritative health information sources: the Centers for Disease Control and Prevention, National Institutes of Health, major research universities, national voluntary health associations (the survey included American Diabetes Association as an example), and responders’ primary health care providers. The answer options ranged on a 5-point Likert scale, from *don’t trust at all* to *trust completely*. The questions were developed based on a literature review of trust in health care and biomedicine [52]. The internal consistency of the health authority trust items, measured by Cronbach α, was 0.64 and considered acceptable [50].

### Data Collection and Preparation

The survey data were collected using Qualtrics XM, a widely used survey software. As a quality control measure, we removed responses that were submitted multiple times (those containing near-identical narrative answers) and responses that were completed in less than 20 minutes. The cutoff was determined empirically by the authors by completing the task as rapidly as possible while viewing the entire video and giving meaningful answers. The resulting data set included 150 responses. One of the participants completed the main information evaluation task only; another completed all the tasks except for the scientific literacy survey, which the participant left partially incomplete. The remaining 148 responses were complete. Missing data for the 2 partial responses were imputed from the means of the corresponding variables.

### Data Analysis

For quantitative analysis, we used multiple-choice data that did not require coding. Scores for each survey were obtained as a simple count of the correct answers. Open-ended responses were reviewed carefully and used to provide illustrations and outline the narrative context of the quantitative data. SPSS (IBM) was used for quantitative data analysis. All subsections of the Results section, except for the last one, titled “Relationship between willingness to share the video and information competencies,” report descriptive statistics illustrated by narrative examples. The last subsection reports the results of the inferential analysis using the statistical methodology described in this subsection.

## Results

### Willingness to Share the Video and Conceptualization of Evidence

Overall, the participants found the video worth sharing. Of the 150 participants, 70% (105/150) were *very likely* (47/150, 31.3%) or *somewhat likely* (58/150, 38.7%) to recommend the video to their respective cousin, whereas only 11.3% (17/150) were *very unlikely* to do that. Those likely to recommend the video put forward several reasons why they found it worth forwarding. Some had to do with their previous beliefs about the effectiveness of dietary supplements and the particular ingredients in the advertised supplement to enhance the immune system.

For example, one participant stated:

I am a firm believer in supplements.

and another wrote:

With ingredients like Zinc and Elderberry, I feel more confident in this pill, as I bombarded myself with elderberry last flu season and it seemed to stop my cold.

Other reasons had to do with the confidence in the wisdom of the crowd and the quality control afforded by the visibility of public opinion in the internet era, “If the quality is not good, many people [would] have complained.” Some expressed trust in the channel or TV show on which the video aired, and with which they had prior familiarity. A number felt that there was no harm in trying something that may potentially be of help (eg, “Since [he] hasn’t recovered for a few weeks, try all of them, maybe it works”). However, many others provided statements that did not include justifications (eg, “It’s a good choice and I think it will work”).

The participants who were unlikely to recommend the video also provided different justifications. Some mentioned the absence of *evidence* of the supplement’s effectiveness or the lack of Food and Drug Administration’s approval. Many were taken aback by the proposed schedule of taking the supplement every hour. Typically, regardless of their willingness to share the video, participants did not provide multiple reasons and did not weigh *pro* and *con* arguments against one another.

Participants were also presented with a number of hypothetical statements that could be viewed as evidence and asked whether these would affect their opinion about the supplement, either positively or negatively (Table 2 contains statements and the results). The large majority responded that their opinion about the supplement would be positively influenced by factors that do not meet the criteria of valid scientific evidence. These included a friend’s account of a positive experience with the supplement (124/150, 82.7% of individuals), high customer ratings on a website of crowdsourced reviews (108/150, 72% of individuals), a survey of supplements consumers done without controls (99/150, 66% of individuals), and a statement on the supplement producer’s site that 9 out of 10 people found the product beneficial (98/150, 65.3% of individuals).

**Table 2:** Likelihood of the following things affecting the opinion about the supplement (N=150).

Statement	Participants, n (%)		
	Positive effect^a^	No effect	Negative effect^a^
You speak to another friend, and she says that she has been taking this product for the past 2 years and has never caught a cold.^b^	124 (82.6)	21 (14)	5 (3.3)
A crowdsourcing review website focused on supplements found that almost every reviewer had positive things to say about the supplement (its average rating being 4.5 out of 5 stars).^b^	108 (72)	29 (19.3)	13 (8.6)
A crowdsourcing review website focused on supplements found that many people were dissatisfied with the supplement (its average rating being 2.3 out of 5 stars).^c^	24 (16)	40 (26.6)	86 (57.3)
A survey of consumers who had been using the product for the last year indicates that 85% of them did not get the flu last year and 15% got the flu.^b^	99 (66)	47 (31.3)	4 (2.6)
On the supplements company’s website, they state that a study found that 9 out of 10 people found the product to be beneficial.^b^	98 (65.3)	48 (32)	4 (2.6)
On the supplements company’s website, a video explains the benefits of the supplement in terms of the biochemistry of how it boosts the immune system.^b^	92 (61.3)	52 (34.6)	6 (4)
This supplement has never been tested in a controlled clinical trial.^c^	20 (13.3)	31 (20.6)	99 (66)
A year later, a review by scientists from the National Institute of Allergy and Infectious Diseases concludes that scientific evidence about the effectiveness of this supplement is inconclusive.^c^	40 (26.6)	37 (24.6)	73 (48.6)
Another year later, a new review by scientists from the National Institute of Allergy and Infectious Diseases concludes that scientific evidence supports the claim that this supplement reduces frequency (how often) and severity (how bad) of respiratory (breathing-related) infections.^b^	127 (84.6)	18 (12)	5 (3.3)

^a^Combines *very likely* and *somewhat likely*.

^b^May be perceived as *in favor* evidence.

^c^May be perceived as *against* evidence.

In explaining their responsiveness to potential influences, many participants stressed the value of their friends’ personal experiences. They described relationships with friends as built on trust, which translated into the information being seen as trustworthy. For example, one participant said, “Personal experience from a trusted person is persuasive.” The term *trust*, mentioned frequently, was described as an assurance of accuracy and objectivity:

I trust what my friends say. So her experience would make me believe in the product’s efficacy more than the information from the morning show guest. I perceive the guest to have a pecuniary interest in sales of the product.

Many respondents also had positive views of reviews on crowdsourcing sites. Although some mentioned possible reasons for skepticism, such as the number of reviews or reviews by bots, many felt reassured by the absence of negative reviews. For example, one respondent wrote, “So many people use it and the effect is very good, so I believe this product has good quality.” However, others preferred to obtain recommendations from their doctors.

In considering a survey that found that 85% of the product’s users did not get the flu in a year, most felt reassured by the number, stating that, in their experience, the number was high enough to suggest that the product was effective. There were also some doubts voiced by participants; for example, “I have a feeling that people who seek out and spend money on supplements like these tend to be healthier in many areas of their lives, so are less likely to get the flu in general.” Responses to testimonies and claims of effectiveness on the company’s site also included a mix of positivity and skepticism. They ranged from “They did research and the results are good, so I think the product quality is good” to “The credibility of research done by his own company is not guaranteed.”

Participants also reacted to the hypothetical scientific evidence. A total of 73 (48.6%) participants said their opinion of the supplement would be negatively affected if a study by the NIAID deemed the evidence of the supplement’s effectiveness inconclusive. Overall, 99 (66%) participants reacted to “This supplement has never been tested in a controlled clinical trial” by stating that this would affect their opinion *very* or *somewhat* negatively. Had a NIAID study found evidence of the supplement’s effectiveness, 127 (84.6%) respondents’ opinions would be positively affected. Overall, many participants expressed a view of science as an important foundation of health-related knowledge. They referred to scientists as having “authority” and “credibility.” One person stated, “Dr. Fauci and the National Institute of Allergy and Infectious Diseases conduct research in a scientific manner and this is very good.” Many expressed trust in NIAID and “national institutions,” “public health agencies,” and “government agencies.” Skeptical voices also existed: “I would still be skeptical, and would want to know about who funded the study, what they found, how much the supplement actually reduced frequency, severity, etc.” Yet others explicitly preferred their own conclusions and experience.

### Information Competencies

#### Information Literacy

For the information literacy measure with a possible range of scores from 0 to 6, participants’ mean score, or the number of correct responses to the multiple-choice questions, was 3.23 (SD 1.72). Table 3 shows the distribution of information literacy scores.

Responses to individual information literacy questions are summarized in Table 4. In evaluating the reliability of information sources, most participants recognized the value of dot-gov and, to a lesser extent, dot-edu domains. For example, in choosing among 3 possible websites in search of “unbiased information” about food to support a child’s immune system, 72.4% (108/149) responders chose a dot-gov site that was “checked by health professionals.” In deciding which site was most likely to provide accurate health information, 59.7% (89/149) chose “an institute run by the US government,” followed by “a support group for patients living with a particular illness” (46/149, 30.9%). In choosing the most reliable site between Harvard Health Publishing website, Healthline website, Medlinx website, and BBC website, a little over half (77/149, 51.7% of respondents) wrote that the Harvard site was the most reliable. When it came to evaluating author credentials as markers of their authority and qualification to write about the immune system, a little over half (76/149, 51%) participants chose an allergist (a physician specializing in immune deficiency disorders) over a food chemist (28/149, 18.8%), a naturopath (27/149, 18.1%), or a health blogger (18/149, 12.1%).

**Table 3:** Distribution of participants’ information literacy scores (N=149).

Score	Participants, n (%)
0	6 (4)
1	19 (12.8)
2	28 (18.8)
3	39 (26.2)
4	18 (12.1)
5	16 (10.7)
6	23 (15.4)

**Table 4:** Information literacy, correct responses (N=149).

Question	Answer options^a^	Correct responses, n (%)
Lisa has a toddler and is looking for a website with unbiased information about food to support her daughter’s immune system. She finds 3 websites. Which of the sites is the best option for Lisa?	The 3 options differ in domains (dot-com, *dot-gov*, dot-info, recency, and background of authors)	108 (72.5)
Which of the following sources’ websites is most likely to provide accurate health information?	Four options ranging from a *US federal institution* to a social media company selling a health app	89 (59.7)
You want to find more information about making your immune system stronger. You type *boost immune system* into Google. From the results of that search, which website is likely the most reliable information source?	*www.health.harvard.edu* www.healthline.com www.medlinx.com www.bbc.com	77 (51.7)
Which of the following authors would be the best qualified to write an article about the immune system?	a food chemist, PhD a naturopath, NMD a food blogger, MA *an allergist, MD*	76 (51)
You go to the mercola.com website, which features health news and articles. The website states that “The entire contents of this website are based upon the opinions of Dr. Mercola, unless otherwise noted.” Does this mean the content has been reviewed by independent medical professionals (eg, qualified doctors, nurses, or other health care providers?)	Yes, definitely *No, not necessarily*	72 (48.3)
The developers of a drink called BoostRx claim that their product increases the effectiveness of the immune system. Which of the additional information below would provide the strongest evidence supporting this claim?	Four options that include *published independent studies*, studies by the developer, advertisements, and purchasers’ reviews	60 (40.3)

^a^Correct answers in italics.

Another information literacy question asked participants to determine whether the content of a health information site was verified by independent reviewers. Despite the explicit statement in the question that “the entire contents” were based on the opinions of the site’s owner, Dr. Mercola, fewer than half (72/149, 48.3%) of the participants chose *no, not necessarily*, whereas the majority (77/149, 51.7%) believed that the site was *probably* verified by independent medical professionals.

The most difficult information literacy question turned out to be the one asking participants to choose a piece of information that would provide the strongest evidence for the claim that a drink called BoostRx increased the effectiveness of the immune system. Only 40.3% (60/149) of respondents correctly chose links to published studies that did not involve developers. A total of 31.2% (47/149) chose reviews by satisfied purchasers, 24.9% (37/149) preferred articles written by one of the developers, and 5 selected advertisements on the developer’s site.

#### Immune System Knowledge

For the measure of immune system knowledge, with possible scores ranging from 0 to 5, the mean score was 2.38 (SD 1.34). The distribution of scores is presented in Table 5.

The question answered correctly by most of the participants was “What do vaccines do?” The majority, 70.5% (105/149), selected “Stimulate the immune system to produce antibodies.” The majority (100/149, 67.1% of participants) also recognized that white blood cells produced antibodies. A little fewer than half (72/149, 48.3%) were able to answer what constituted the first line of defense against microbes (skin). About a third, approximately 36.2% (54/149) correctly selected the answer that described the relationship between antibodies and antigens. Finally, only 16.1% (24/149) recognized that all 3 organs, cells, and chemicals were components of the immune system.

**Table 5:** Distribution of participants’ immune system knowledge scores (N=149).

Score	Participants, n (%)
0	12 (8.1)
1	29 (19.5)
2	38 (25.5)
3	43 (28.9)
4	17 (11.4)
5	11 (7.4)

#### Science Literacy

For the measure of science literacy, with possible scores ranging from 0 to 12, the mean score was 6.57 (SD 2.40). The distribution of the scores is shown in Table 6.

**Table 6:** Distribution of participants’ science literacy scores (N=148).

Score	Participants, n (%)
0	0 (0)
1	0 (0)
2	0 (0)
3	10 (6.8)
4	23 (15.5)
5	29 (19.6)
6	17 (11.5)
7	22 (14.9)
8	13 (8.8)
9	8 (5.4)
10	14 (9.5)
11	11 (7.4)
12	1 (0.7)

The questionnaire included 2 question formats. The first presented an accurate or faulty reasoning statement and asked, “Is this a good scientific argument?” The other format described the design of a study, either well-designed or problematic, and a conclusion, and asked whether, based on the presented information only, any other factors could explain the difference (Table 7). In both cases, participants were less likely to recognize faulty reasoning or design than they were to doubt good logic and well-designed experiments. The most difficult questions turned out to be those that required recognizing potential confounding factors in the research design. Considering base rates and sample sizes also proved challenging (see Table 7 for examples).

**Table 7:** Selected questions requiring recognizing potential confounds in research design (N=148).

Question	Answer^a^	Correct, n (%)
Researchers want to study how noise affects task performance. They randomly put participants into 2 groups. Females make up 35% of the first group and 75% of the second group. Participants in the first group complete a moderately difficult task in a quiet room. Participants in the other group do the same task in a noisy room. Researchers say that any differences in performance between the groups will be because of the noise. Based on this information only, do you see any other factors that may explain the difference?	*Yes* No	47 (31.8)
*This year, there were 100,000 more cases of adolescent depression diagnosed in the US than last year. Thus, adolescent depression in the US is on the rise.* Is this a good scientific argument?	Yes *No*	63 (42.6)
Researchers in a cancer clinic test a new drug in 12 patients with a rare cancer. None of the patients experience any dangerous side effects. The researchers concluded that the drug is safe. Based on this information only, do you see any factors in the design that make you less confident about the researchers’ interpretation of their findings?	*Yes* No	58 (39.2)
*Many people who take multi-vitamins do not catch colds frequently. Thus, taking multi-vitamins prevents colds.* Is this a good scientific argument?	Yes *No*	77 (52)

^a^Correct answers in italics.

#### Trust

On the measure of interpersonal trust, deciding whether *most people can be trusted* where 1 meant *you can’t be too careful* and 5 *most people can be trusted*, the majority of respondents leaned toward trusting people. Although most participants trusted major biomedical research and policy organizations and the health care establishment, a not so small minority was skeptical of them (Table 8). The mean health authority trust score was 20.08 (SD 2.81) out of 25.

**Table 8:** Trust in health authority (N=149).

	Participants, n (%)				
	1 (do not trust at all)	2	3	4	5 (trust completely)
Majority of people^a^	11 (7.4)	7 (4.7)	47 (31.5)	68 (45.6)	16 (10.7)
National Institutes of Health	1 (0.7)	11 (7.4)	16 (10.7)	55 (36.9)	66 (44.3)
Centers for Disease Control and Prevention	1 (0.7)	9 (6)	21 (14.1)	68 (45.6)	50 (33.6)
Your primary doctor or health care provider	0 (0)	3 (2)	33 (22.1)	71 (47.7)	42 (28.2)
A national health association, such as American Diabetes Association	1 (0.7)	3 (2)	39 (26.2)	61 (40.9)	45 (30.2)
A major university that conducts biomedical research	3 (2)	5 (3.4)	38 (25.5)	65 (43.6)	38 (25.5)

^a^For this question, answer options are 1=cannot be too careful and 5=can be trusted.

### Relationship Between Willingness to Share the Video and Information Competencies

For the inferential analysis, we were interested in the impact of information literacy, science literacy, knowledge of the immune system, interpersonal trust, and trust in health authority on the likelihood of recommending the video.

As a first step in the analysis, we looked at pairwise correlations among the independent variables of interest. The variables were highly correlated (Table 9).

**Table 9:** Correlations among independent variables (with significance level; N=150).

Independent variable			Information literacy		Science literacy		Immune system knowledge		Interpersonal trust		Health authority trust
**Information literacy**											
	*r*	1.000		0.505^a^		0.286^a^		−0.390^a^		0.143	
	*P* value	N/A^b^		<.001		<.001		<.001		.08	
**Science literacy**											
	*r*	0.505^a^		1.000		0.357^a^		−0.228^a^		0.175^c^	
	*P* value	<.001		N/A		<.001		.005		.03	
**Immune system knowledge**											
	*r*	0.286^a^		0.357^a^		1.000		−0.157		0.112	
	*P* value	<.001		<.001		N/A		.06		.17	
**Interpersonal trust**											
	*r*	−0.390^a^		−0.228^a^		−0.157		1.000		0.235^a^	
	*P* value	<.001		.005		.06		N/A		.004	
**Health authority trust**											
	*r*	0.143		0.175^c^		0.112		0.235^a^		1.000	
	*P* value	.08		.03		.17		.004		N/A	

^a^Significant at the .001 level (2-tailed).

^b^Not applicable.

^c^Significant at the .05 level (2-tailed).

As interventions often target a single variable, we wanted to compare effect sizes of single predictor models. To do this, we conducted 5 single-predictor regression analyses of the independent variables on the 4-level *likelihood of recommending the video*. To correct for multiple hypotheses, the significance of the models was assessed at *P*<.01. The data are summarized in Table 10.

**Table 10:** Comparing single predictor models.

Independent variable	F test (*df*)	*P* value	Adjusted *R*^2a^	Standardized coefficient β	Unstandardized coefficient *B* (CI; SE)	*t* test (*df*)	*P* value
Information literacy	31.24 (1,148)	<.001^b^	0.17	−0.417	−0.236 (−0.320 to −0.153; 0.042)	−5.59 (148)	<.001^b^
Interpersonal trust	29.04 (1,148)	<.001^b^	0.16	0.405	0.393 (0.249 to 0.538; 0.073)	5.38 (148)	<.001^b^
Science literacy	11.65 (1,148)	<.001^b^	0.07	−0.270	−0.110 (−0.174 to −0.046; 0.032)	−3.41 (148)	<.001^b^
Immune system knowledge	6.17 (1,148)	.01^c^	0.03	−0.200	−0.146 (−0.262 to −0.030; 0.059)	−2.48 (148)	.01^c^
Health authority trust	2.40 (1,148)	.12	0.01	0.126	0.044 (−0.012 to −0.101; 0.029)	1.55 (148)	.12

^a^Reflects proportion of variance accounted by the model.

^b^Statistically significant at *P*<.01.

^c^Approaches significance at *P*<.01.

The analysis shows that, as single predictors, information literacy had the largest effect size, predicting the largest amount of variance in the dependent variable (17%). Although only 22% (5/23) of the participants with the highest possible information literacy score of 6 said they would share the video, 95% (18/19) with a score of 1 would share it. The percentage of participants wishing to share the video increased sharply at the information literacy score of 5 (11/16, 69%) and continued to increase (41/57, 72% at the scores of 4 and 3, 25/28, 89% at the score of 2).

The effect size of information literacy was closely followed by that of interpersonal trust (accounting for 16% of the variance). Science literacy as a single predictor accounted for 7% of the variance in the dependent variable and immune system knowledge for 3%. Trust in health authority had no significant effect as a single predictor.

To compare the strength of associations, we calculated correlations of each predictor with the dependent variable and then used the Fisher r-to-z transformation. The analysis revealed that the top 3 predictors, namely information literacy, interpersonal trust, and science literacy, were not statistically different in the magnitude of their association with the likelihood of sharing the video.

The effects of the variables were in the expected directions: higher information literacy, science literacy, and immune system knowledge scores were associated with a lower likelihood of recommending the video. Higher interpersonal trust was associated with a greater likelihood of recommending the video.

Due to multiple correlations leading to expectation of shared variance, we also performed a linear regression with all 5 independent variables in the single model. Multicollinearity diagnostic tests were performed and did not raise concerns about violations of assumptions for regression (all VIFs≤1.48). The model was statistically significant, *F*_1,144_=10.60; *P*<.001, and accounted for 24% of the variance in the likelihood of recommending the video. In the final model with all the predictors entered, information literacy (2-tailed t_149_=−3.26; *P*=.001) and interpersonal trust (t_149_=2.85; *P*=.005) were statistically significant; trust in health authority approached significance (t_149_=1.72; *P*=.09); immune system knowledge and science literacy were not significant with the other factors in the model.

The overall analysis suggests that information literacy, science literacy, immune system knowledge, and interpersonal trust were related to participants’ willingness to share the video; the impact of trust in health authority was marginal. Moreover, the correlation among the predictors and different significance of the variables in the single predictor and multiple predictor models suggest the possibility of mediation. For example, it is possible that information literacy mediated the impact of scientific literacy variables, with science literacy variables influencing judgment not directly, but by affecting information literacy.

## Discussion

### Overview

This study confirms that people are vulnerable to web-based misinformation and are likely to propagate it by sharing it with others [8-19]. In evaluating claims, they are often influenced by the information that science and information professionals within the normative health care paradigm do not consider supporting evidence. Examples include hearsay, majority opinions, or statements by those with conflicts of interest. The study also shows that information literacy, science literacy, and health domain knowledge are challenging competencies. None of this is surprising.

### Theoretical and Practical Contributions

The study’s major theoretical and practical contribution to the field is in demonstrating that greater information literacy and science literacy are associated with lesser vulnerability to misinformation and lesser propensity to share it. Although the field of eHealth recognizes these literacies, they have received little attention in empirical research [53]. Although the relationship is complex, and vulnerability to misinformation is affected by a host of interacting factors, programs that target information literacy and understanding of science hold promise for helping individuals and communities. Of these 2 factors, information literacy is easier to address in informal educational settings, such as libraries, community organizations, and health clinics. Science literacy, on the other hand, is primarily developed over a period of years in a science classroom. Still, the two correlate and likely influence one another, pointing to the value of a collaborative conversation among school science educators, information professionals, and health professionals.

Another contribution of this study to the field is providing a starting point for building instruments for assessing information literacy and science literacy as they pertain to health information and information behaviors. The instruments developed for this study build on existing tools developed for related purposes. Although developing robust psychometric instruments requires multiple rounds of item testing and adjustment that are beyond the scope of this study, the internal consistencies of our initial instruments pose them as a feasible foundation. Further work is needed to sharpen and validate them for use in consumer health information contexts.

Finally, unlike much research into the public’s reaction to web-based health information [8,10,14], this study focuses on a YouTube video, rather than web text. YouTube videos as an information source pose a unique challenge to consumers: their authorship and ownership are often harder to establish, whereas auditory information and the absence of hyperlinks make verifying authority and fact checking more challenging. In addition, existing web-based information evaluation criteria have not been optimized for assessing the quality of videos [54,55]. At the same time, videos are an attractive format to watch and easy to share, and studying consumer behavior in sharing online health videos is important.

### The Issue of Trust

This discussion would be incomplete without addressing the issue of trust and the need for further research into the relationship between trust and vulnerability to health misinformation. One important area of trust that merits further investigation is trust in science [52]. In designing our science literacy instrument, we chose to focus on knowledge about science (eg, understanding scientific methods and argumentation), rather than on trust in science as an enterprise. In evaluating potential supporting evidence for the claims made in the video, our participants made many positive claims about science. For example, clinical trials of the effectiveness of the supplement were the type of evidence that raised confidence in the supplement for the greatest number of participants. In future research, we intend to explore trust in science more closely.

The 2 kinds of trust that were investigated in this study, interpersonal trust and trust in health authority, also merit further investigation. It was unexpected that trust in health authority was only marginally associated with vulnerability to misinformation and in only one of the models. This may be due to the specific health information domain addressed by the study, dietary supplements for strengthening the immune system. Scientific data addressing the impact of supplements on health are very complex and do not easily translate into evidence-based public health messaging. In other areas, such as attitudes toward vaccination, trust in health authority and vulnerability to misinformation may have a stronger association.

Interpersonal trust’s association with vulnerability to health misinformation posits a philosophical challenge. In a way, it is not surprising that people who are less trusting in general are also more skeptical of overgeneralized, inflated promises of cures, unsupported by research evidence. At the same time, social science research does not equate trust with gullibility [46] and generally views higher interpersonal trust as a characteristic of healthy functional societies. Institutional trust also positively correlates with trust in societal institutions [45] and should be expected to correlate with trust in science and health care establishments. To the best of our knowledge, no studies have conducted an in-depth investigation of different types of trust related to the spread of misinformation on the internet. The topic, however, is important, especially as it relates to misinformation around COVID-19 infodemic, and merits further studies.

### Implications for Education and Consumer Health Informatics

Efforts to help the public navigate and share health information in the era of social media can be of 2 kinds. The first kind involves programs, activities, and materials that target individuals’ information competencies. Such programs can occur in a variety of settings, focusing on helping individuals analyze the characteristics of information sources, presentation, and content. Our study suggests that such interventions have promise. At the same time, information and science literacy develop over time, growing with many educational and personal experiences, so easy fixes are unlikely. Moreover, these competencies do not tell the full story when it comes to predicting information behavior. As an illustration, in our sample, many participants with high competencies scores expressed an intent to share the video. The science literacy background section of this paper discusses how people often make choices that appear inconsistent with their scientific knowledge, driven by considerations of group identity [38] or fast reactive responses [40].

The second kind of approach to supporting the public involves leveraging technologies. Examples of technologies can vary, from fact-checking sites to machine learning misinformation detection tools [56]. The development of such tools is important because it alleviates the burden of being vigilant for consumers and also reduces the cognitive load while processing complex information. This entails less time commitment than educational efforts.

However, technology alone will not solve the problem of misinformation. The challenge of evaluating information authority applies to a fact-checking tool as much as it applies to a YouTube video selling dietary supplements. Moreover, trust in information and information providers is critical for the acceptance of any information technology. Helping individuals recognize web-based health misinformation is a complex, multifaceted enterprise that should involve the collaborative efforts of researchers, technology developers, librarians, educators, and community outreach specialists.

### Limitations and Directions for Future Research

As this study was conducted with a small nonrepresentative sample, it does not provide information on the general picture of the propensity to share health misinformation on the internet, as well as related literacies, in the population. Moreover, the study used a hypothetical scenario and could not draw on the complex social factors that affect decisions to share information in real life (eg, approval and status seeking). Supplementing this study with research into real-world health information sharing decision making will provide more nuanced data for future understanding of the issue.

Furthermore, no validated psychometric instruments exist currently for assessing information literacy and science literacy in the context of web-based health information behavior. Although our research instruments were designed with care, the formal process of designing, testing, adjusting, and validated psychometric tools was outside the scope of our study. Although the internal consistency data of our instruments were acceptable, a higher reliability is expected of formal instruments. We hope that as attention to information literacy, science literacy, and trust grows, such robust instruments will emerge. At present, our results should be interpreted with the understanding that they are affected by our specific selection of questions and answer options in our instruments. As the lower internal consistency of instruments makes the effects more difficult to detect, the sizes of the effects found here may be greater in future studies.

Finally, our study involved a survey methodology with a primarily quantitative focus, an approach that works better for establishing relationships than for describing them in depth. An interview study probing participants’ reasoning would provide a nuanced richness of information on this important topic.

## Appendix

Multimedia Appendix 1 All instruments.

## Abbreviations

NIAID: National Institute of Allergy and Infectious Diseases
NNLM: National Network of the Libraries of Medicine
RML: Regional Medical Library
TOSLS: Test of Scientific Literacy Skills
