# Supplementary material for: Factors Influencing Willingness to Share Health Misinformation Videos on the Internet: Web-Based Survey
Source: J Med Internet Res. 2021 Dec 9;23(12):e30323. doi: 10.2196/30323 (PMC8704117; doi:10.2196/30323)
Supplement: Multimedia Appendix 1 [file jmir_v23i12e30323_app1.docx]

**Appendix A: All instruments**

**Online health information literacy task**

**Imagine the following scenario:**
Your cousin had a bad case of the flu last year and did not fully recover for several weeks. In recent years, your cousin has frequently suffered with colds and occasionally, the flu. A friend told you about how certain supplements can help rev up your immune system. That friend recommends the following video.

After viewing the video, answer the following questions.

1. After reviewing the video, how likely are you to recommend the supplement to your cousin? Please, explain your decision in a few sentences.
   1. very unlikely
   2. somewhat unlikely
   3. Somewhat likely
   4. Very likely
2. Please describe a segment or segments from the video that you found convincing. Please, explain why in a few sentences.
3. Please describe a segment or segments from the video that raised concerns. Please, explain why in a few sentences.
4. You speak to another friend and she says that she has been taking this product for the past two years and has never caught a cold. How likely is that to affect your opinion about the supplement? Please, explain why in a few sentences.
   1. very likely, negatively
   2. somewhat likely, negatively
   3. no effect
   4. somewhat likely, positively
   5. very likely, positively
5. A crowd-sourcing review website focused on supplements found that almost every reviewer had positive things to say about the supplement (its average rating being 4.5 out of 5 stars). How likely is that to affect your opinion about the supplement? Please, explain why in a few sentences.
   1. very likely, negatively
   2. somewhat likely, negatively
   3. no effect
   4. somewhat likely, positively
   5. very likely, positively
6. A crowd-sourcing review website focused on supplements found that many people were dissatisfied with the supplement (its average rating being 2.3 out of 5 stars). How likely is that to affect your opinion about the supplement? Please, explain why in a few sentences.
   1. very likely, negatively
   2. somewhat likely, negatively
   3. no effect
   4. somewhat likely, positively
   5. very likely, positively
7. A survey of consumers who had been using the product for the last year indicates that 85% of them did not get the flu last year and 15% got the flu. How likely is that to affect your opinion about the supplement? Please, explain why in a few sentences.
   1. very likely, negatively
   2. somewhat likely, negatively
   3. no effect
   4. somewhat likely, positively
   5. very likely, positively
8. On the supplements company’s website, they state that a study found that 9 out of 10 people found the product to be beneficial. How likely is that to affect your opinion about the supplement? Please, explain why in a few sentences.
   1. very likely, negatively
   2. somewhat likely, negatively
   3. no effect
   4. somewhat likely, positively
   5. very likely, positively
9. On the supplements company’s website, a video explains the benefits of the supplement in terms of the biochemistry of how it boosts the immune system. How likely is that to affect your opinion about the supplement? Please, explain why in a few sentences.
   1. very likely, negatively
   2. somewhat likely, negatively
   3. no effect
   4. somewhat likely, positively
   5. very likely, positively
10. This supplement has never been tested in a controlled clinical trial. How likely is that to affect your opinion about the supplement? Please, explain why in a few sentences.
    1. very likely, negatively
    2. somewhat likely, negatively
    3. no effect
    4. somewhat likely, positively
    5. very likely, positively
11. A year later, a review by scientists from the National Institute of Allergy and Infectious Diseases concludes that scientific evidence about the effectiveness of this supplement is inconclusive. How likely is that to affect your opinion about the supplement? Please, explain in a few sentences.
    1. very likely, negatively
    2. somewhat likely, negatively
    3. no effect
    4. somewhat likely, positively
    5. very likely, positively
12. Another year later, a new review by scientists from the National Institute of Allergy and Infectious Diseases concludes that scientific evidence supports the claim that this supplement reduces frequency (how often) and severity (how bad) of respiratory (breathing-related) infections. How likely is that to affect your opinion about the supplement? Please, explain in a few sentences.
    1. very likely, negatively
    2. somewhat likely, negatively
    3. no effect
    4. somewhat likely, positively
    5. very likely, positively

**Demographic questions**

1. What is your age:

- 18-29
- 30-49
- 50-64
- 65+

2. What is your highest education level:

- High school or less
- Some college
- College graduate
- Postgraduate degree

3. Do you think of yourself as:

- Male
- Female
- Gender nonconforming, neither exclusively male nor female
- Additional gender category, or other
- Decline to answer

4. Do you identify as:

- American Indian or Alaska Native
- Asian
- Black or African American
- Hispanic or Latino
- Native Hawaiian or Other Pacific Islander
- White
- Other
- Decline to answerer

5. In what state do you live:

6. What best describes the area where you live?

- Urban
- Rural
- Suburban

**Information literacy survey (correct answers in bold)**

1. You want to find more information about making your immune system stronger. You type “boost immune system” into Google. From the results of that search, which website is likely the most reliable information source?
   1. **www.health.harvard.edu**
   2. www.healthline.com
   3. www.medlinx.com
   4. [www.bbc.com](http://www.bbc.com)
2. You go to the mercola.com website which features health news and articles. The website states that “The entire contents of this website are based upon the opinions of Dr. Mercola, unless otherwise noted.” Does this mean the content has been reviewed by independent medical professionals (e.g., qualified doctors, nurses or other healthcare providers?)
   1. ___ Yes, definitely
   2. ___ **No, not necessarily**
3. Lisa has a toddler and is looking for a website with unbiased information about food to support her daughter’s immune system. She finds 3 websites. Which of the sites is the best option for Lisa?
   1. Website A ends in .com. This website sells natural foods. There is no information about when the content was written or who wrote it.
   2. Website B ends in .info. The website content is written by a mother of 3 children and the information about food was last updated in March 2017.
   3. **Website C ends in .gov. The website is maintained by a department of the U.S. federal government. The content has been checked by health professionals and the information about food was last updated in August 2020.**
4. Which of the following authors would be the best qualified to write an article about the immune system?
   1. Maria Alonso, PhD, a food chemist
   2. Indira Acharya, NMD., a naturopath
   3. Sven Larsson, MA, a health blogger
   4. **Pat Petersen, MD, an allergist**
5. Which of the following sources’ websites is most likely to provide accurate health information?
   1. **An institute run by the U.S. federal government.**
   2. A support group for patients living with a particular illness.
   3. A company selling medical devices.
   4. A social media company selling an app to connect patients with other patients.
6. The developers of a drink called BoostRx claim that their product increases effectiveness of the immune system. Which of the additional information below would provide the strongest evidence supporting this claim?
   1. Reviews by satisfied purchasers of the product.
   2. Links to published scientific articles written by one of the developers
   3. **Links to published scientific studies of BoostRx, not conducted or sponsored by the developers.**
   4. Advertising on BoostRx’s website.

**Trust survey**

1. Generally speaking, would you say that most people can be trusted, or that you can't be too careful in dealing with people? Please rate your response on the scale from 1 to 5, where 1 means you can't be too careful and 5 means most people can be trusted.

2. How much confidence do you have in the following institutions’ ability to provide accurate, reliable health information? Please, rate your trust in them on the scale from 1 to 5, where 1 is you don’t trust them at all and 5 is you trust them completely.

- Centers for Disease Control and Prevention, CDC
- National Institutes of Health, NIH
- Your primary doctor or healthcare provider
- A major university that conducts biomedical research
- A national health association, such as American Diabetes Association

**Immune system knowledge survey (correct answers in bold)**

1. Which of the following are components of the immune system? (check all that apply)
   1. **Organs**
   2. **Cells**
   3. **chemicals**
2. What is considered the first line of defense against microbes?
   1. Lungs
   2. B-cells and T-cells
   3. Antibodies
   4. **Skin**
3. White blood cells produce:
   1. Antigens
   2. **Antibodies**
   3. B-cells
   4. Stem cells
4. What is the relationship between antibodies and antigens?
   1. Antibodies activate antigens, which then destroy invaders
   2. **Antibodies latch onto antigens and destroy them**
   3. Antibodies and antigens work together to destroy invaders
   4. Antibodies produce adaptive immunity, and antigens produce innate immunity
5. What do vaccines do?
   1. **Stimulate the immune system to produce antibodies**
   2. Destroy viruses and bacteria
   3. Replace natural immunity with vaccine-induced immunity
   4. Create a mild version of an illness

**Science literacy survey (correct answers in bold)**

1. *Many people who take multi-vitamins do not catch colds frequently. Thus, taking multi-vitamins prevents colds.* Is this a good scientific argument?
   1. *Yes*
   2. ***No***
2. *Scientists genetically engineered a strain of mice that lacked a certain gene. These mice could not reproduce. Scientists then re-introduced the gene back into the mice. Now the mice could reproduce. Thus, this gene is critical for mouse reproduction.* Is this a good scientific argument?
   1. ***Yes***
   2. *No*
3. *Nuplazid is a drug approved for treatment of Parkinson’s disease. 34% of caregivers of people living with Alzheimer’s believe that Nuplazid may also alleviate symptoms of Alzheimer’s, because both diseases involve death of neurons. Thus, Nuplazid can be prescribed for Alzheimer’s.* Is this a good scientific argument?
   1. *Yes*
   2. ***No***
4. *This year, there were 100,000 more cases of adolescent depression diagnosed in the US than last year. Thus, adolescent depression in the US is on the rise.* Is this a good scientific argument?
   1. Yes
   2. **No**
5. *A study randomly assigned 2,000 people who wanted to quit smoking into two groups. People in the first group participated in a weekly support group. People in the second group participated in weekly sessions of a cognitive behavioral therapy (CBT) smoking cessation program. At the end of 6 months, more people from the second group haven’t smoked for a month. Thus, CBT is more effective for quitting smoking than participation in a support group*. Is this a good scientific argument?
   1. **Yes**
   2. No

**Take a look at the following descriptions of research studies. Evaluate the reasonableness of the conclusions. Only use the information that is given below, without assuming anything else.**

1. Researchers want to study how noise affects task performance. They randomly put participants into two groups. Females make up 35% of the first group and 75% of the second group. Participants in the first group complete a moderately difficult task in a quiet room. Participants in the other group do the same task in a noisy room. Researchers say that any differences in performance between the groups will be because of the noise. Based on this information only, do you see any other factors that may explain the difference?
   1. **Yes**
   2. No
      1. If yes, what is it?
2. Researchers want to study religious beliefs of students in U.S. universities. They send a survey to a random selection of 500 freshmen at a small private university in Tennessee. To the researchers, the findings represent religious beliefs of the U.S. university students. Based on this information only, do you see any factors in the design that make you less confident about the researchers’ interpretation of their findings?
   1. **Yes**
   2. No
      1. If yes, what is it?
3. To evaluate the effect of a new diet program, researchers compare weight loss between participants randomly assigned to treatment (diet) and control (no diet) groups, while controlling for average daily exercise and pre-diet weight. At the end, they ascribe differences between weight loss between the two groups to the program. Based on this information only, do you see any factors in the design that make you less confident about the researchers’ interpretation of their findings?
   1. Yes
   2. **No**
      1. If yes, what is it?
4. Researchers compared the effectiveness of two arthritis medications. The first medication was tested in patients in a rural clinic in Midwest. Most participants were farmers who lived with their spouses and children. The second medication was tested in a nursing home in large city in the Northeast. Both groups had the same number of participants, who were following the same medication schedule. The group that received the first medication had better response, so the researchers concluded that the first medication is more effective. Based on this information only, do you see any factors in the design that make you less confident about the researchers’ interpretation of their findings?
   1. **Yes**
   2. No
      1. If yes, what is it?
5. Researchers want to know whether stress contributes to sugar consumption. They conduct a large study in which, over a period of 5 years, they collect information on the diet and lifestyles of 100,000 people living in different regions and communities in the United States. In addition to diet and stress level questions and measures, researchers also collect information on family structure, work and leisure, income, education, and a number of other social and demographic factors. The researchers concluded that there is a connection between stress and sugar consumption. Based on this information only, do you see any factors in the design that make you less confident about the researchers’ interpretation of their findings?
   1. Yes
   2. **No**
      1. If yes, what is it?
6. Researchers in a cancer clinic test a new drug in 12 patients with a rare cancer. None of the patients experience any dangerous side effects. The researchers concluded that the drug is safe. Based on this information only, do you see any factors in the design that make you less confident about the researchers’ interpretation of their findings?
   1. **Yes**
   2. No
      1. If yes, what is it?
7. Scientists conducted a systematic review of 10 years of published studies in peer-reviewed journals that investigated the impact of CalmX on anxiety. The review included 214 studies, the methodological quality of which was judged by the systematic reviewers as ranging from "acceptable" to "high". Of these studies, two-thirds were funded by government institutions and one- third by the pharmaceutical industry. 186 studies found that CalmX reduced anxiety in the study participants, while 24 showed no effect. Based on this information, which of the following is most likely:
   1. CalmX is an effective drug for treating anxiety in most people.
   2. CalmX is not effective for treating anxiety. Some other factors, for which researchers did not control, interfered and made a difference. Researchers wrongly attributed the effect of that other factor to anxiety.
   3. CalmX is not effective for treating anxiety in most people. The designs of the studies were good, but, by pure chance, participants in the study had a very statistically unusual response.
   4. Any of the above is equally likely
